# Dry-Coated Live Viral Vector Vaccines Delivered by Nanopatch Microprojections Retain Long-Term Thermostability and Induce Transgene-Specific T Cell Responses in Mice

**DOI:** 10.1371/journal.pone.0067888

**Published:** 2013-07-09

**Authors:** Frances E. Pearson, Celia L. McNeilly, Michael L. Crichton, Clare A. Primiero, Sally R. Yukiko, Germain J. P. Fernando, Xianfeng Chen, Sarah C. Gilbert, Adrian V. S. Hill, Mark A. F. Kendall

**Affiliations:** 1 Delivery of Drugs and Genes Group (D2G2), Australian Institute for Bioengineering and Nanotechnology, The University of Queensland, Brisbane, Queensland, Australia; 2 The Jenner Institute, The University of Oxford, Oxford, United Kingdom; 3 Diamantina Institute, The University of Queensland, Brisbane, Queensland, Australia; 4 Vaxxas Pty Ltd, Australian Institute for Bioengineering and Nanotechnology, Brisbane, Queensland, Australia; Mayo Clinic, United States of America

## Abstract

The disadvantages of needle-based immunisation motivate the development of simple, low cost, needle-free alternatives. Vaccine delivery to cutaneous environments rich in specialised antigen-presenting cells using microprojection patches has practical and immunological advantages over conventional needle delivery. Additionally, stable coating of vaccine onto microprojections removes logistical obstacles presented by the strict requirement for cold-chain storage and distribution of liquid vaccine, or lyophilised vaccine plus diluent. These attributes make these technologies particularly suitable for delivery of vaccines against diseases such as malaria, which exerts its worst effects in countries with poorly-resourced healthcare systems. Live viral vectors including adenoviruses and poxviruses encoding exogenous antigens have shown significant clinical promise as vaccines, due to their ability to generate high numbers of antigen-specific T cells. Here, the simian adenovirus serotype 63 and the poxvirus modified vaccinia Ankara – two vectors under evaluation for the delivery of malaria antigens to humans – were formulated for coating onto Nanopatch microprojections and applied to murine skin. Co-formulation with the stabilising disaccharides trehalose and sucrose protected virions during the dry-coating process. Transgene-specific CD8^+^ T cell responses following Nanopatch delivery of both vectors were similar to intradermal injection controls after a single immunisation (despite a much lower delivered dose), though MVA boosting of pre-primed responses with Nanopatch was found to be less effective than the ID route. Importantly, disaccharide-stabilised ChAd63 could be stored for 10 weeks at 37°C with less than 1 log_10_ loss of viability, and retained single-dose immunogenicity after storage. These data support the further development of microprojection patches for the deployment of live vaccines in hot climates.

## Introduction

The overall success of a vaccination campaign is measured by the protective efficacy of a vaccine and the population coverage achieved. One of the factors limiting access to most licensed vaccines is the requirement for their delivery by hypodermic needles. The logistical disadvantages of needle delivery are most pertinent in under-resourced healthcare settings, often coinciding with the heaviest malaria burdens [1]. Since candidate malaria vaccines have indicated only modest levels of protective efficacy in clinical trials to date [2], the number of individuals with direct access to a future vaccine must be high in order to achieve herd immunity. Removing the barriers to vaccine access, such as the requirement for needle delivery, is expected to improve vaccine distribution and uptake.

Desirable attributes of a needle-free ‘patch’ for vaccine delivery include: (1) a small size for ease of distribution; (2) simple and accurate administration; (3) projections sharp enough to penetrate through the *stratum corneum,* though; (4) short enough not to stimulate dermal pain receptors or pose a waste disposal problem; (5) a simple vaccine-loading procedure without requirement for denaturing temperatures or changes in pH; (6) efficient release of coated material into the skin; and (7) stability of coated vaccine patches at high temperatures over time. Perhaps most importantly, for such an intervention to replace well-established practices in the clinic, immunogenicity must be at least comparable to existing needle methods. The Nanopatch was designed with these desirables in mind, with the aim of circumventing current logistical challenges presented by needle delivery of liquid vaccine.

Upon Nanopatch application, silicon microprojections coated with vaccine deliver their payload directly into the vicinity of a network of skin antigen-presenting cells [3]. Vaccine diffuses into micro-channels penetrating into the viable epidermis (VE) and dermis [4]. Nanopatch microprojections are of extremely high density (>20,000 cm^−2^), distinguishing them from other reported microneedle patch technologies with densities of <5000 cm^−2^ [4]. Using a spring-loaded applicator for dynamic delivery [5], we have delivered a broad range of microprojection-coated vaccines by Nanopatch, including plasmid DNA, virus like particle, split virion, recombinant protein and killed virus [3], [6]–[9]. In the case of a split virion influenza vaccine, Nanopatch-induced Haemagglutinin Antigen (HA)-specific antibody responses were equivalent to those induced by intramuscular injection with only 1/100^th^ of the vaccine dose [3]. We have also recently demonstrated a similar ‘dose-sparing’ effect with respect to T cell responses against Nanopatch-delivered recombinant protein antigen, with and without co-formulated adjuvant [10].

Recombinant viral vectors are among the most promising platforms for the development of new T cell-inducing vaccines. These non-replicating, live viruses (commonly delivered by intradermal injection, ID) are particularly immunogenic when a different vector is used to prime responses as to boost them, e.g. adenovirus prime, poxvirus (e.g. modified vaccinia Ankara, MVA) boost, where both vectors express a common transgene antigen [11]. Removal of genes through attenuation allows for insertion of exogenous genes encoding malaria, influenza, tuberculosis or HIV antigens [12]. MVA has an established safety and immunogenicity profile from use in human clinical trials [13], [14]. Simian adenoviruses (e.g. Chimpanzee serotype 63, ChAd63) are now commonly used in place of human serotypes, such as AdHu5, to circumvent the problem of pre-existing anti-vector immunity [15]. Simian vectors have shown to be comparable to AdHu5 in cellular tropism, receptor utilisation and immunogenicity in mice, and ChAd63 has been determined safe and immunogenic in recent trials in macaques and humans [16]–[19].

Stable conformation of viral vector protein structures is essential to ensure infection of host cells, and hence expression of transgene. Though lyophilisation extends the shelf life of licensed live vaccines, refrigerated transport and storage of both lyophilised vaccine and diluent cannot be currently circumvented, and potency and safety declines rapidly after reconstitution [20]. Incorporation of a simple, needle-free delivery method with improved vaccine thermostability would be ideal for the delivery of live vaccines in regions experiencing high temperatures and unreliable power supplies, such as sub-Saharan Africa – where 91% of global malaria deaths occurred in 2010 [21]. The glass-forming disaccharides trehalose (hereafter ‘TH’) and sucrose (‘SC’) are commonly used in the stabilisation of biological products due to their protective effects upon protein structures during temperature changes and desiccation. This is likely due to the tendency of these sugars to form an amorphous immobilising solid glass upon removal of water [22]. A combination of TH and SC has previously demonstrated successful long-term thermostabilisation of lyophilised adenovirus and MVA vaccines over a range of temperatures [23]. Therefore, we hypothesised that the protective properties of TH and SC would allow stabilisation of these viral vectors during removal of water during our standard Nanopatch coating procedure (previously described [7]).

The present study aimed to investigate the utility of microprojection patches for the delivery of liveChAd63 and MVA viral vectors encoding the malaria antigens ME-TRAP (Multiple Epitope string fused to *Plasmodium falciparum* TRAP) and PbCSP (*Plasmodium berghei* circumsporozoite protein) [24], [25]. The excipients used in Nanopatch coating formulations were found to have no effect on the ability of either virus to infect cells; however, viral viability was significantly compromised during dry-coating onto microprojections. Despite this, viral viability was retained when TH and SC were added into both formulations, in accordance with our hypothesis. Importantly, vaccine antigens encoded by Nanopatch-coated ChAd63 and MVA retained CD8^+^ T cell immunogenicity in mice, comparable to standard ID needle injection after a single immunisation. However, boosting of primed responses with Nanopatch was ineffective. Whilst TH+SC stabilisation was essential for ChAd63 immunogenicity, it was not required for priming of responses by MVA. Finally, we demonstrated that Nanopatches coated with TH+SC-stabilised ChAd63 could be stored for 10 weeks at 37°C with only 0.2 log_10_ loss in viability and retained the ability to induce cellular responses. These data support further development of micro-projection patches for improved provision of live viral vaccines to countries with high environmental temperatures, and for improved vaccination logistics, uptake and compliance.

## Results

### Nanopatch Microprojections Breach the Stratum Corneum and Penetrate into the Viable Epidermis and Dermis

**Figure 1:**
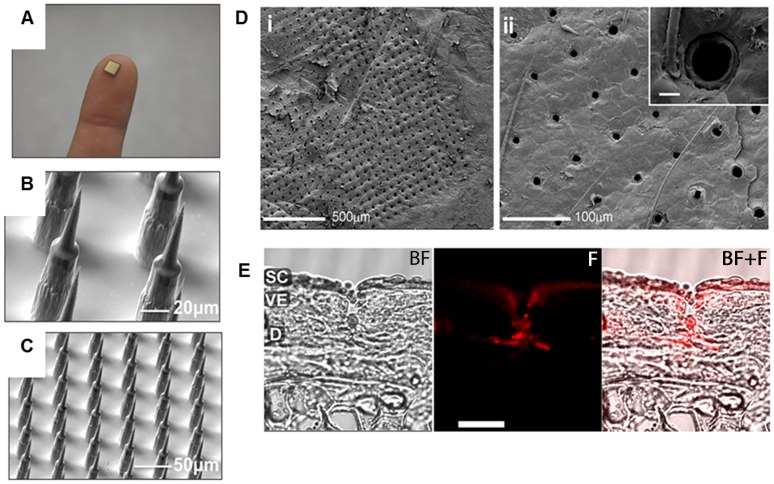
Imaging of skin penetration by Nanopatch microprojections. (A) The size of a single Nanopatch relative to a forefinger. (B+C) SEM images of microprojection morphology after dry-etch fabrication. (D) Representative cryo-SEM images of the mouse ear skin surface following application of a single Nanopatch. D(i) shows a far field view of the corner of the patched area, with micro-channel openings characteristic of microprojection penetration, adjacent to unbroken skin. D(ii) shows perforated area in higher magnification, with a single micro-channel next to a hair follicle inset. Scale bar inset = 10 µm. (E) Representative micrographs of ear tissue sections following delivery of Nanopatch coated with a fluorescent dye. BF: brightfield image, F: fluorescence image, BF+F: both brightfield and fluorescent images overlaid. SC = *stratum corneum*; VE = viable epidermis; D = dermis.

**Fig. 1A** is a photograph of a single Nanopatch shown relative to the size of a forefinger. Each Nanopatch consists of a 16 mm^2^ silicon base with 3364 microprojections (21, 025 cm^−2^). The SEM images shown in **Fig. 1B** **+C** illustrate the morphology of the Nanopatch microprojections used throughout these studies. Firstly, to demonstrate that Nanopatch microprojections breach the skin’s surface, Nanopatches were dynamically delivered to the ears of mice using a mechanical applicator [5]. Cryo-SEM imaging of the mouse ear skin surface revealed widespread and consistent penetration across the patched area. The bottom right corner of the patched area is visible in the representative image in **Fig. 1D** **i**, adjacent to areas with no evidence of *stratum corneum* breaching. Penetration was efficient and localised, with >95% of microprojections breaching the surface, evidenced by the creation of openings with diameters consistent with those of microprojection shafts (**Fig. 1D** **ii**). A single micro-channel is shown in high magnification in **Fig. 1D** **ii, inset**. To image the depth of penetration, a lipophilic fluorescent dye was coated onto microprojections and delivered to mouse ear skin, enabling visualisation of micro-channels created. Representative confocal micrographs (**Fig. 1E**) demonstrate dye delivery into a single micro-channel penetrating through the VE into the dermis, with some lateral diffusion into dermal tissue at the tip. Measurements given are based on those previously reported [26].

### Viral Vector Viability is Maintained during Formulation and Dry-coating, when Formulated with TH and SC

**Figure 2:**
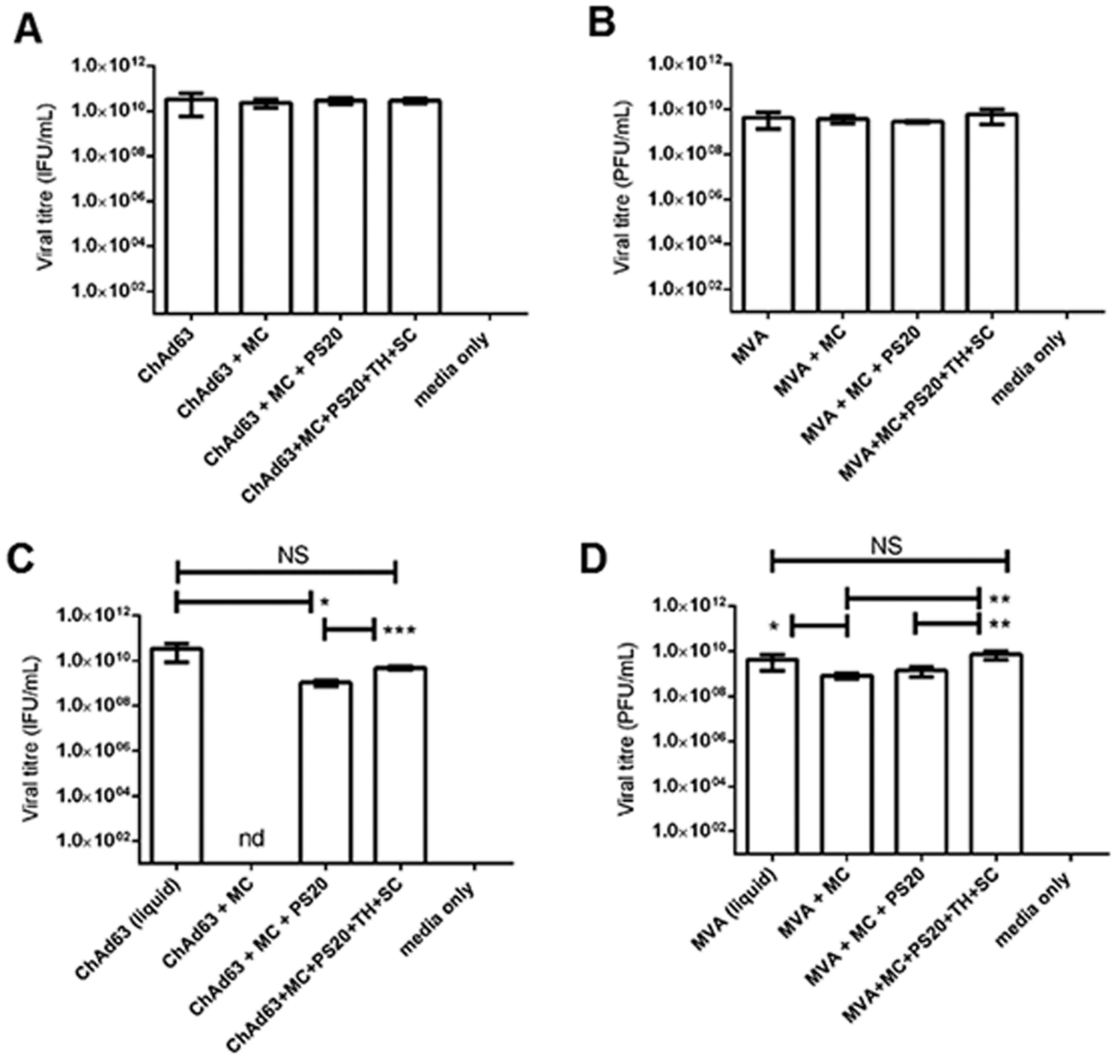
Viral viability throughout formulation and during Nanopatch dry-coating. (A+B) ChAd63.ME-TRAP (1×10^9^ VP) and MVA.GFP (1×10^7^ PFU) were mixed with combinations of MC and PS20, with or or without the disaccharides TH+SC (10% ^w^/_v_ each sugar). Formulations were added to DF-1 cell monolayers (A; MVA, n = 4) or HEK-293A cells (B; ChAd63, n = 5) to evaluate viral titre, which was compared to unformulated virus. (C+D) Formulations containing ChAd63.ME-TRAP (C; 1×10^9^ VP) and MVA.GFP (D; 1×10^7^ PFU) were coated onto Nanopatch and immediately eluted into D-MEM. Eluates (n = 4/5) were added to cell monolayers in infectivity assays as before. Eluted viral titres were compared against unformulated, liquid virus. Negative control wells contained D-MEM only. NS = not significant. nd = no data. IFU = infectious units, PFU = plaque forming units.

The glass-forming disaccharides trehalose and sucrose have been demonstrated to improve viral vector recovery following slow desiccation [23]. Consequently, we hypothesised that TH+SC would convey protective effects on live viral vectors during Nanopatch formulation and dry-coating processes. Firstly, we examined whether the standard coating excipients MC (1% ^w^/_w_) and PS20 (0.01% ^v^/_v_), or the disaccharides TH+SC (10% ^w^/_v_ each sugar), had a destabilising effect upon ChAd63 or MVA in formulation. Coating formulations containing ChAd63.ME-TRAP (**Fig. 2A**) or MVA.GFP (**Fig. 2B**) were assessed for the ability of each virus to infect cells *in vitro*. Each formulation tested demonstrated similar infectivity with titres equivalent to unformulated vaccine (**Fig. 2A**, P = 0.81; **Fig. 2B**, P = 1.18). Therefore, none of the coating excipients used (at the concentrations chosen) had a significant destabilising effect upon either virus when mixed together. We next hypothesised that viral viability would be compromised during removal of water as part of the dry-coating process, but that TH+SC would protect virions during desiccation. Nanopatches were coated with vector-containing formulations either with or without TH+SC. There was a reduction in recovered viral titre of both ChAd63 (**Fig. 2C**, P = 0.03) and MVA (**Fig. 2D**, P = 0.03) following dry-coating. However, when TH+SC were included, there was no detectable reduction in viability comapred to liquid vaccine. Indeed, significant improvements were observed compared to formulations not containing TH+SC (**Fig. 2C**, P = 0.002 and **Fig. 2D**, P = 0.0003). These data indicate a protective role for TH and SC during the dry-coating of both ChAd63 and MVA vaccines.

### Microprojection-coated Viral Vector Vaccine Immunogenicity in Prime Boost Immunisation Schedules

**Figure 3:**
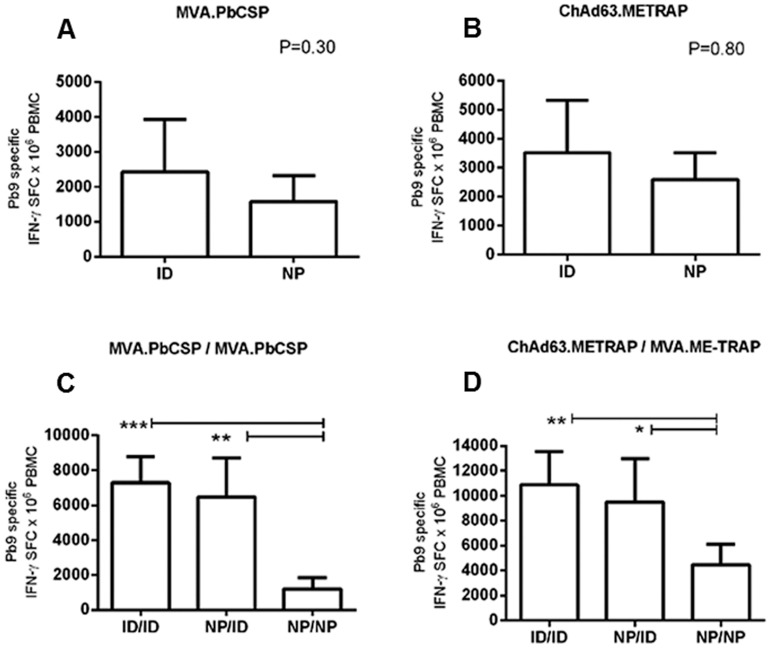
CD8^+^ T cell immunogenicity of Nanopatch-delivered viral vector vaccines in prime boost schedules. Mice (n = 5/6) were primed with 5×10^7^ PFU MVA.PbCSP (no TH or SC) (A) or 5×10^9^ VP ChAd63.ME-TRAP +10% ^w^/_v_ TH+SC (B), either by coated Nanopatch or ID injection. Two weeks post-MVA.PbCSP priming, a boost immunisation of MVA.PbCSP was given and 8 weeks after ChAd63.METRAP a boost immunisation of MVA.ME-TRAP (no TH or SC) was given (dose 5×10^7^ PFU, given either ID or by Nanopatch). One week post-MVA (A) or 3 weeks post-ChAd63 (B) or 2 weeks post-boost (C+D), blood was taken for analysis of Pb9-specific IFN-γ secreting cells. SFC = spot forming cells. PBMC = peripheral blood mononuclear cells.

**Figure 4:**
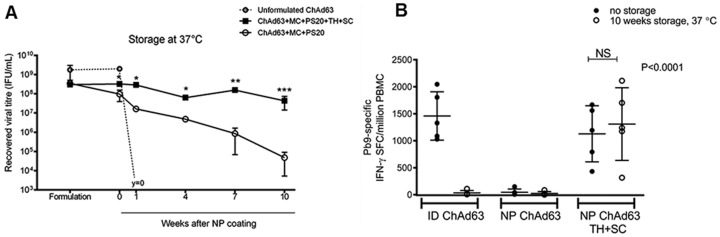
Longer-term thermostability mediated by formulation of ChAd63 vaccine with TH+SC. (A) 5×10^9^ VP ChAd63.ME-TRAP were formulated ± TH+SC and used to coat Nanopatches (n = 4). Coatings were immediately eluted from Nanopatches (‘week 0’) or stored in sealed tubes at 37°C for 1–10 weeks. Hexon immunoassays were carried out at various time-points post-coating to determine recovered infectious viral titre. Liquid, unformulated ChAd63.ME-TRAP was also stored under the same conditions and assayed alongside Nanopatch eluates. (B) CD8^+^ T cell immunogenicity following storage. ChAd63-coated Nanopatches were stored as above for 10 weeks at 37°C. Stored and freshly coated Nanopatches were used to immunise mice (5×10^9^ VP dose). Stored/freshly diluted vaccine (no disaccharaide) was administered ID to control mice. Immunogenicity was ascertained after 3 weeks by IFN-γ ELISPOT. NS = not significant.

Having demonstrated that viral vectors remain viable after Nanopatch coating, we investigated their Pb9-specific immunogenicity in mice when delivered in prime boost schedules (**Fig. 3**). Homologous prime boost regimens such as MVA/MVA are less immunogenic than heterologous regimens (ChAd63/MVA), due in part to the induction of anti-vector immunity after the prime, which inhibits secondary expansion upon boost [27]. Consequently, we investigated both the highly immunogenic ChAd63.ME-TRAP/MVA.ME-TRAP (currently under clinical evaluation) and the less immunogenic MVA.PbCSP/MVA.PbCSP pre-clinical regimen. Preliminary data indicated that whilst TH+SC (optimally, at a concentration of 10% ^w^/_v_) were required for the induction of immune responses by single-dose ChAd63, their inclusion was not essential for the induction of responses following MVA immunisation (**Supporting Figure S1**). Therefore, for assessment of immunogenicity in prime-boost schedules, we included 10% ^w^/_v_ TH+SC in ChAd63 Nanopatch coatings only. **Fig. 4** presents cellular immune responses generated after prime immunisation (**Fig. 3A** **+B**) and after both prime and boost immunisations (**Fig. 3C** **+D**). After a single immunisation (**Fig. 3A** **+B**), Nanopatch-delivered TH+SC-stabilised ChAd63.ME-TRAP and MVA.PbCSP (without TH+SC) were both immunogenic in mice, generating CD8^+^ T cell responses similar to those induced ID. Though the mean Nanopatch-delivered MVA.PbCSP response was lower than ID –1584±733 compared to 2418±1520 SFC/million cells, no statistically significant differences were detected between groups (**Fig. 3A**, P = 0.30; **Fig 3B**, P = 0.80). These data demonstrate that Nanopatch dry-coated vaccines are equally as able to prime responses as is ID delivery of liquid vaccine. Using a radio-labelled tracer protein (^14^C-ovalbumin), the proportion of vector-containing material transferred from the Nanopatch into the ear was determined to be 14.5% ±4.0 (of the total amount coated) for MVA-containing coatings, and 1.3% ±1.1 for ChAd63-containing coatings (**Supporting Table S1**). However, after a boost vaccination (**Fig. 3C** **+D**), significant differences were detected between groups with both regimes (**Fig 3C**, P = 0.001; **Fig 3D**, P = 0.002). Responses primed by Nanopatch and boosted ID were similar to those after ID delivery of both immunisations, using both schedules (though responses were not improved upon those limited by effects of anti-vector immunity in MVA/MVA). Nanopatch boosting of Nanopatch-primed responses was not effective, with significantly lower post-boost responses than both ID/ID (**Fig 3C**, P = 0.0003; **Fig 3D**, P = 0.0009,) and Nanopatch/ID (**Fig. 3C**, P = 0.005; **Fig. 3D**, P = 0.013). These results indicate that, within these particular vaccine regimes, whilst Nanopatch is efficient at inducing primary responses, it is not able to boost responses that are already primed.

### Longer-term Thermostability of ChAd.ME-TRAP Vaccine Mediated by Trehalose and Sucrose

In addition to protection during removal of water, the disaccharides TH and SC have been reported to stabilise lyophilised and reconstituted AdHu5 at temperatures up to 45°C for 6 months [23]. As a thermostable vaccine patch would be highly desirable in the context of malaria immunisation, we investigated the ability of the TH+SC mixture to mediate thermostabilisation of ChAd63 (**Fig. 4**). Studying the stability of MVA and AdHu5 over time, Alcock *et al* noted a proportional relationship between loss of AdHu5 infectious titre and immunogenicity, but a 4 log_10_ drop in MVA titre resulted in only a 100-fold reduction in response [23]. Therefore, the adenoviral vector was selected for stability studies as it was deemed the more difficult of the two vectors to retain long-term stability and immunogenicity. Live ChAd63 recovered from Nanopatch coatings was titrated after various periods in storage at 37°C. Unformulated vaccine was rendered completely non-infectious after only one week. Of Nanopatch coatings, more viable virus was recovered after formulation with TH+SC, immediately after coating (‘week 0’) and at each time point after storage (**Fig. 4A**, P = 0.0007). In agreement with the result in **Fig. 2**, no difference in viral titre between groups was detected in formulation (prior to dry-coating). Stabilised vaccine demonstrated only a 0.2 log_10_ mean total loss in viability over the course of 10 weeks of storage, significantly improved over unstabilised vaccine at time points 1 (**Fig. 4A**, P = 0.01), 4 (P = 0.008), 7 (P = 0.0006) and 10 weeks (P = 0.0005). In contrast, the mean total loss of viability of unstabilised vaccine over the 10 week observation was approximately 3.5 log_10_. Disaccharide-stabilised Nanopatches stored at 37°C for 10 weeks were used to immunise mice to determine their ability to induce CD8^+^ T cell responses (**Fig. 4B**). These stored Nanopatches induced cellular responses after a single ChAd63 immunisation that were comparable to those induced by freshly coated patches, and fresh ID vaccine (no significant differences were detected between these groups). However, unstabilised Nanopatch-coated ChAd63 was not immunogenic, either before or after storage (a similar trend is observed in **Supporting Figure S1**). As expected, storage of liquid vaccine (without TH+SC) did not induce a response after ID delivery. These data demonstrate that in addition to providing protection during the dry-coating procedure, TH+SC also mediate longer-term thermostability of Nanopatch-coated live ChAd63 vaccine.

## Discussion

Our data provide a basis for the further development of microprojection patches for the delivery of live, viral vaccines. The key finding of this study is that viral vectors remained viable and immunogenic, not only in formulation and throughout a drying process necessary for Nanopatch coating, but also throughout 10 weeks of storage at 37°C, when ChAd63 was mixed with the stabilising disaccharides TH and SC. Additionally, both freshly coated and stored Nanopatches were able to induce CD8^+^ T cell immune responses in mice.

The first challenge in the transcutaneous delivery of biomolecules is overcoming the *stratum corneum*, permeable only to molecules <500 Da [28]. Vaccines based on complex viruses are several orders larger than this, so this barrier must be breached to allow delivery of viral vectors into the skin. We demonstrated efficient *stratum corneum* perforation and creation of micro-channels into the VE and dermis when microprojections were applied dynamically, in line with previous results [5] (**Fig. 1**). These sites are rich in Langerhans cells and langerin^+^ dermal dendritic cells, both of which been implicated in the priming of CD8^+^ T cell responses [29], [30]. The application condition used is consistent with what has been previously reported in mice [5], showing that dynamic application of the Nanopatches to skin achieves controlled, discrete penetration causing some initial inflammation that is rapidly resolved [31].

Next, we investigated the effect of Nanopatch coating procedures upon live viral vectors. Others in the microneedle field report that formulation of AdHu5 and MVA with Carboxymethyl Cellulose (CMC) is associated with no recovery of adenoviral, and negligible recovery of MVA (when MVA was formulated with CMC +15% ^w^/_v_ TH, compared to TH alone) [32]. A similar negative effect on microneedle-coated Bacillus Calmette-Guerin has been noted [33]. Elsewhere, CMC was found to protect inactivated influenza virus against destabilisation caused by the effect of high concentration of co-formulated TH, and also that the inclusion of surfactant was detrimental to vaccine stability [34]. Here, we report no reduction of viral titre of either ChAd63 or MVA when formulated with either our standard viscosity enhancer Methyl Cellulose (MC; lacking the terminal COO- group of CMC, indicating a possible effect of the chemistry of reactive groups within excipients for mediating instability) or a low concentration of the surfactant PS20 (**Fig. 2**).

We tested the hypothesis that viral viability would be compromised by the dry-coating process itself, but that formulation with TH and SC could protect against this. Our hypothesis was supported by a previous report from Alcock *et al* demonstrating that TH and SC stabilised AdHu5 and MVA through slow desiccation and deposition onto a filter membrane, remaining stable for up to 6 months at temperatures up to 45°C. After reconstitution, viral titre was completely recovered and transgene immunogenicity in mice was retained [23]. Whilst these advances remove the costly refrigeration requirement in the clinic or field, needles were required for delivery. In agreement with the findings of Alcock *et al,* we found that recovered ChAd63 titre was significantly decreased following drying compared to liquid vaccine, unless mixed with TH+SC (**Fig. 2**). Additionally, **Supporting Figure S1** suggests that ChAd63 immunogenicity was dependent upon inclusion of these disaccharides. In contrast, though MVA viability was similarly affected by drying (which could be similarly restored by TH+SC), these sugars were found to be dispensable for the induction of immune responses (**Fig. 4** **+S1**). Similarly, Alcock *et al* noted that whereas losses in infectious titre were proportional to AdHu5 immunogenicity, a 4 log_10_ drop in MVA titre resulted in only a 100-fold reduction in response [23]. This suggests that MVA is more inherently stable than ChAd63, the immunogenicity of which is more sensitive to environmental stresses. This could be due the adaptation of poxviruses for complete desiccation in dermal scabs [35], whereas the natural route of infection for adenovirus is mucosal [36]. This previously reported observation together with our observation that disaccharide stabilisation was not required for immunogenicity of MVA, informed our decision to take the less stable of the two vectors, ChAd63, into a longer-term thermostability study.

Maintenance of an uninterrupted cold chain is estimated to increase the overall cost of vaccination by 14% [37]. The WHO stability requirement for live measles vaccine is that viral loss must not exceed 1 log_10_ viral particles over the course of 7 days at 37°C [38]. Others have shown that when this vaccine is dip-coated onto microneedles, viability is retained for 30 days at 22°C, though was reduced by over 3.5 log_10_ when stored at 37°C [39]. Our formulation exceeds the WHO requirement by 9 weeks, and improves upon the latter study by demonstrating successful storage of live vaccine at an elevated temperature of 37°C. It is interesting that viral titre was retained even with unstabilised ChAd63 for 10 weeks at 37°C, albeit with a greater loss in infectivity compared to stabilised virus (**Fig. 4A**). We have previously shown that unstabilised Nanopatch-coated inactivated influenza vaccine stored at 22°C is able to induce anti-HA titres after 6 months of storage [40]. This suggests that the process of dry-coating itself has stabilising effect, likely through the rapid removal of water by nitrogen jet. Elsewhere, TH has been used to stabilise non-live vaccines coated onto microneedle patches [32], [41]–[44]. When inactivated virus and VLP influenza vaccines were coated onto 700 µm microneedles using a dip-coating method, the haemagglutinin inhibition ability of antibodies induced was found to decrease (attributed to VLP aggregation), but could be restored with co-formulation with 15% ^w^/_v_ TH, impacting on vaccine immunogenicity and protective efficacy [42]–[45]. Inactivation was found to occur primarily within 30 minutes following coating, associated with residual moisture during gradual air drying, and then during storage of coated patches at 4–37°C for 28 days. TH was able to partially stabilise inactivated influenza vaccine throughout 28 days of storage at 4°C and 25°C, but after storage at 37°C HA-activity (used as a measure of stability) dropped below 20% [43]. In contrast to this dip-coating and gradual drying method, our dry-coating method actively removes all moisture within 5 minutes. Recently, a dissolving microneedle modality (44×1500 µm microneedles/patch) for the delivery of live AdHu5 vectors encoding ovalbumin or HIV-1 gag antigen was described [46]. Vaccine was formulated with 8% ^w^/_v_ sodium CMC and 30% ^w^/_v_ SC and stored for up to 1 month at room temperature (25°C), before reconstitution and subcutaneous injection of mice. CD8^+^ T cell immunogenicity was retained following storage, though immunogenicity of patches stored for 1 month at this temperature was not presented. Dried dissolving patches demonstrated similar single-dose cellular responses to needle-based routes [46].

Elsewhere, when aqueous MVA.PbCSP was delivered by a range of microneedle arrays, the induction of Pb9-specific CD8^+^ T cells was found to be equivalent or superior to ID immunisation, depending upon specific array parameters [47]. This group has also proposed spray-coating formulations and parameters for AdHu5 and MVA, using 15% ^w^/_v_ TH, and demonstrate similar single-dose CD8^+^ T cell and antibody responses to ID injection [32]. Here, responses following a single Nanopatch immunisation to both vaccines were similar to ID delivery, despite a very low efficiency of vaccine transfer (**Supporting Table S1**). It is acknowledged that as viral infection and transgene expression cannot directly be correlated with availability of soluble ^14^C-ovalbumin tracer used in the measurement of transfer efficiency, therefore, the dose delivered by Nanopatch and the dose delivered by ID injection were not matched in these experiments. Nevertheless, despite a significantly lower dose delivery, Nanopatch responses following a single immunisation were equivalent to ID. It should be noted that delivery efficiency values were much lower than with previous reports of Nanopatch delivery of non-viral vaccines (∼30% e.g. [3], [9]) and are likely a result of high viscosity of viral vector preparations. Nanopatch boosting of responses was unsuccessful. We believe this is due to a threshold dose delivery requirement for boosting (but not for priming, *unpub. obs*.) and is a result of the low transfer efficiency of these vaccines – for the particular configuration we have tested here in this study. Indeed, it can be argued that this Nanopatch configuration tested on mice – with low delivery efficiency – is not suitable for replacing ID injection of these viral vectors in prime boost schedules. It is worth noting, however, that when this Nanopatch delivery configuration is applied to other types of vaccines; effective boosting was achieved. Examples include anti-HSV-2 IgG [6] and OVA-specific T cell responses [10]. Elsewhere, advances in the Nanopatch coating process have increased the transfer efficiency of coated influenza vaccine to ∼32% [40] and indeed ∼82% [48]. We anticipate that this will lead to a stronger Nanopatch boost, possibly inducing similar levels of immunogenicity when both prime and boost are delivered by Nanopatch, as when they are delivered ID.

If this could be achieved, the next step could be translating the Nanopatch to become a clinical device for improved malaria vaccination. This work would build upon the practical design of the Nanopatch for simple, low-cost vaccination in the field (these features within the context of improving the reach of vaccines in low-resource regions are detailed in [40]). Examples include: applying the Nanopatch to skin with a simple spring-based applicator, achieving uniform and consistent penetration within the skin and good local tolerability ([5]; in mice); and, packaging of the device to ensure the Nanopatch projections are not exposed in handling – mitigating the scope for cross-contamination and needle-stick injury.

### Conclusion

Removing the significant costs associated with liquid, needle-based delivery of vaccines will allow greater access to immunisation services, particularly within poorly resourced health systems. Here, we demonstrate effective stabilisation of live viruses throughout dry-coating processes and that virus retains immunogenicity both immediately after coating and after storage at elevated temperature. In a prime-boost immunisation regime, inferior Nanopatch boosting was demonstrated compared to higher-dose intradermal boosting. We speculate that this could be improved by a higher Nanopatch dose delivery of live viral vectors, achieved by using more advanced vaccine coating approaches that are currently under development.

## Materials and Methods

### Ethics Statement

Experiments were approved by the UK Home Office (license numbers 30/7793 and 30/2414), the University of Oxford Animal Care and Ethical Review Committee and the University of Queensland Anatomical Biosciences Ethics Committee (AEC AIBN 020/10 and 520/80).

### Microprojection Patches

Deep Reactive Ion Etch fabrication of Nanopatches has been described [4]. Microprojections (21, 025 cm^−2^) used were of total length 110 µm (70 µm shaft +40 µm tip, **Fig. 1**). Details of patents filed on the Nanopatch technology are given in **Supporting Table S2**.

### Vaccines and Coating Formulations

The ChAd63 vector was provided by Okairos, Italy. MVA expressing the *Plasmodium berghei* circumsporozoite (PbCSP) or Green Fluorescent Protein (GFP), and ChAd63 and MVA expressing antigen ME-TRAP were constructed, propagated and titrated as described elsewhere [18], [24], [49]. Vaccines were mixed with 1% ^w^/_w_ methyl cellulose (MC; Fluka, Australia.) and 0.01% ^v^/_v_ polysorbate 20 (PS20; Sigma-Aldrich, Australia, with or without D-(+)-trehalose dehydrate (Sigma-Aldrich, Australia) and sucrose dehydrate (Fluka, Australia) dissolved in sterile water. Nanopatches were dry-coated with vaccine as previously described [7].

### Mice and Immunisations

Female BALB/c mice (6–8 weeks) were from Harlan (UK) Monash University or the Animal Resource Centre (Australia). Female C57BL/6 mice were from University of Queensland. Mice were anaesthetised by injection of ketamine hydrochloride (80 mg/kg; Fort Dodge Animal Health, UK/Troy Laboratories, Australia) and medetomidine/xylazine hydrochloride (10 mg/kg; Pfizer, UK/Troy Laboratories, Australia). Anti-sedan® (1 mg/kg; Pfizer) was given to reverse the effects of anaesthesia. ID delivered vaccines were diluted in endotoxin-free PBS and 25 µL injected into each ear pinna. Vaccine-coated Nanopatches were mechanically-applied to ear skin at a velocity of 1.96±0.12 ms^−1^ with a peak transient application force of approximately 42 N in 10 ms^−1^ [5]. Nanopatches were removed after 5 minutes of application. For immunogenicity studies, one Nanopatch was delivered to each ear.

### Scanning Electron Microscopy (SEM)

SEM of uncoated Nanopatches was performed on a JEOL NeoScope JSM 5000 microscope (45° tilt, 15 kV). For imaging of the skin surface after application, Nanopatches were applied to C57BL/6 mouse ears (as described above, with the modification that Nanopatches remained *in situ* after application). Immediately after application ears were excised and snap-frozen in a slush liquid nitrogen solution. Nanopatches were removed from tissue immediately prior to SEM imaging at −180°C.

### Confocal Microscopy

Vybrant® DiD (2% ^v^/_v_, Molecular Probes Inc., USA) was coated onto microprojections and delivered to mouse ear skin. Whole ears were excised and cryo-preserved in liquid nitrogen. Using a cryostat (Leica Microsystems, Germany), 10 µm sections of whole ear were taken from a number of areas across the patched area and imaged using a confocal microscope (LSM510META, Carl Zeiss Inc., Germany).

### Viral Viability Assays

Vaccine was mixed with coating excipients as described above. Formulations or material immediately eluted from coated patches into complete Dulbecco’s Modified Eagle Medium (D-MEM, **Supporting Methods File S1**) were used in viral titration assays (ChAd63– hexon immunostaining; MVA – plaque assay). For method see **Supporting Methods File S1.**

### Assessment of Immunogenicity

Mice were immunised using established prime boost protocols [50], [51]. At various time points post-immunisation, Peripheral Blood Mononuclear Cells (PBMC) isolated from blood or splenocytes were re-suspended in complete Minimal Essential Media (MEM, **Supporting Methods File S1**). Cells were incubated with peptide Pb9 (SYIPSAEKI, MHC class I, an epitope within both PbCSP and ME-TRAP antigens) in an IFN-γ ELISPOT protocol [52]. For method see **Supporting Methods File S1**.

### Thermostability Studies

Nanopatches were coated with ChAd63.ME-TRAP) ±10% ^w^/_v_ TH and SC and stored for 1–10 weeks in sealed tubes at 37°C at a constant humidity of 11% [40]. At various time points, coatings were eluted into 1 mL D-MEM. Recovered virus was titrated by hexon immunostaining assay or used to immunise mice for assessment of immunogenicity (**Supporting Methods File S1**).

### Statistical Analysis

Analyses were performed using one-way or two-way Analysis of Variance (ANoVA) with subsequent unpaired t tests. Graphpad Prism v5 (www.graphpad.com) or R (http://www.r-project.org) were used. Significance was assigned to P<0.05 (*P<0.05, **P<0.005, ***P<0.001). Error bars signify standard deviation of the mean.

## Supporting Information

Figure S1 **Effect of TH+SC upon the induction of CD8^+^ T cell responses.** Mice (n = 3) were immunised ID or by Nanopatch (NP) with either: (A) 5×10^9^ VP ChAd63.ME-TRAP, or (B) 1×10^7^ PFU MVA.PbCSP. Vaccines were mixed with MC and PS20 in standard concentrations and with TH+SC in concentrations of each sugar of 5% w/v (grey bars) or 10% w/v (black bars), or without sugars (Nil, open bars). The same concentrations of TH+SC were mixed with PBS which was injected ID (‘Mock’). Three weeks (ChAd63) or one week (MVA) following a single immunisation, immune responses were measured by Pb9 re-stimulation of splenocytes and IFN-γ ELISPOT (**Supporting Methods File S1**). This preliminary experiment aimed to select the concentration of TH+SC for use in immunogenicity studies. Nanopatches were coated with ChAd63 (A) or MVA (B) either without TH+SC, or with TH+SC at concentrations of 5% ^w^/_v_ total disaccharide (equating to approximately 0.15M, as per [23]) or 10% ^w^/_v_ total disaccharide (maximum concentration possible without negatively affecting coating morphology). Results indicated that of the two concentrations, optimal ChAd63 immunogenicity was induced with addition of 10% ^w^/_v_ TH+SC_,_ whereas inclusion of TH+SC to MVA vaccines was not a requirement for the induction of a response. Therefore, 10% ^w^/_v_ TH+SC was taken forward as the optimal concentration for ChAd63 stabilisation.
(DOCX)

Table S1 **Proportions of vaccine-containing coated material delivered to the mouse ear skin upon NP application.** NP (n = 5) were coated with formulations containing MVA (2.5×107 PFU or ChAd63 (2.5×10^9^ VP) with or without the addition of 10% ^w^/_v_ TH+SC. 4 nCi of the radio label ^14^C-Ovalbumin (^14^C-OVA). Coated NP were delivered to mice and β particle emission detected from excised ears, swabs used to remove material on the ear surface and used NP. Proportions of ^14^C-OVA detected by scintillation assay present within the excised ear, as a percentage of the total ^14^C-OVA (ear+swab+NP) are given in the table. The summed counts were similar to elution controls – NP coated and immediately eluted into PBS (not shown). The delivery efficiencies of ChAd63.ME-TRAP and MVA.PbCSP were similar (ChAd63; 16.5% ±2.8, MVA; 14.5% ±4.0).When TH+SC were added into the formulation, the delivery efficiency was significantly reduced (P<0.0001), though scintillation counts were significantly higher than the limit of detection (averaged counts from 10 vials of PBS only, not shown). Delivery efficiencies of vectors with TH+SC addition were 1.3% ±1.1 (ChAd63) and 0.08% ±0.06 (MVA). The addition of TH+SC significantly increased the viscosity of the coating solution (empirical observation). Consequently, we speculate that the low delivery efficiency of virus when formulated with TH+SC is due to imperfect coating morphology mediated by increased viscosity of coating solution.
(DOCX)

Table S2 **Table listing all patents filed on the Nanopatch technology and details of author involvements.**
(DOCX)

Methods File S1 **Additional materials and methods used within these studies.**
(DOCX)
