# Supplementary figures and images for: Dry-Coated Live Viral Vector Vaccines Delivered by Nanopatch Microprojections Retain Long-Term Thermostability and Induce Transgene-Specific T Cell Responses in Mice
Source: PLoS One. 2013 Jul 9;8(7):e67888. doi: 10.1371/journal.pone.0067888 (PMC3706440; doi:10.1371/journal.pone.0067888)

# Supporting Figure S1 – Pearson et al

**
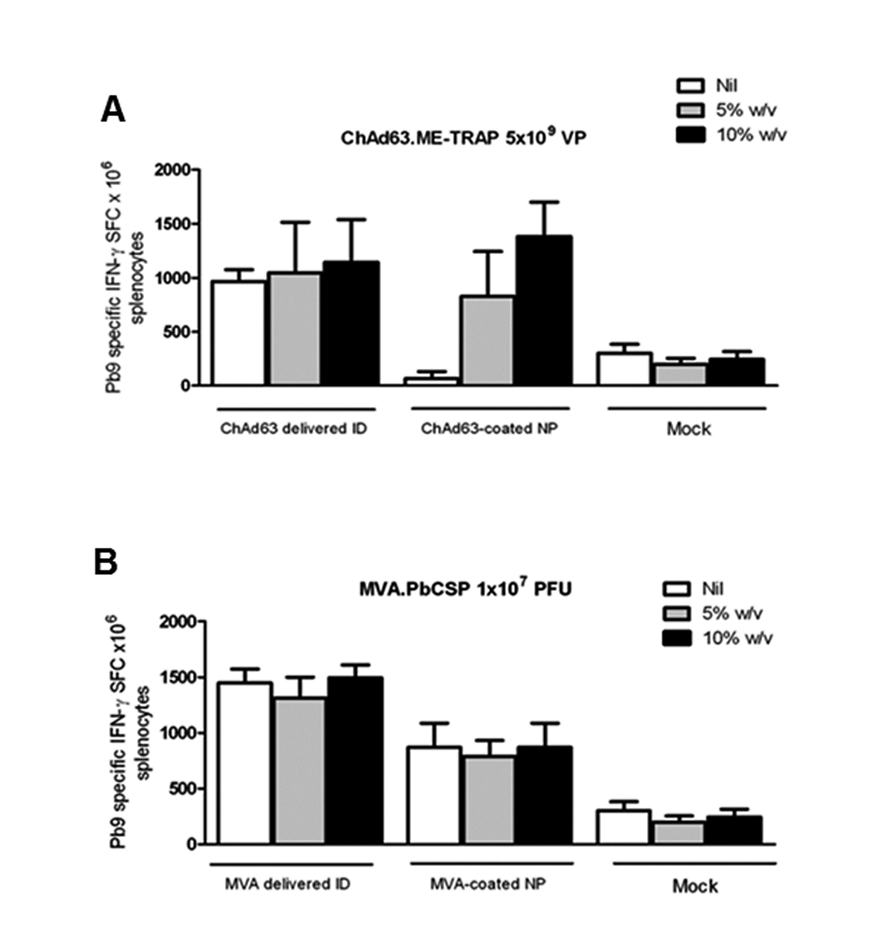
**

Supplement: Figure S1 — Effect of TH+SC upon the induction of CD8+ T cell responses. Mice (n = 3) were immunised ID or by Nanopatch (NP) with either: (A) 5×10^9^ VP ChAd63.ME-TRAP, or (B) 1×10^7^ PFU MVA.PbCSP. Vaccines were mixed with MC and PS20 in standard concentrations and with TH+SC in concentrations of each sugar of 5% w/v (grey bars) or 10% w/v (black bars), or without sugars (Nil, open bars). The same concentrations of TH+SC were mixed with PBS which was injected ID (‘Mock’). Three weeks (ChAd63) or one week (MVA) following a single immunisation, immune responses were measured by Pb9 re-stimulation of splenocytes and IFN-γ ELISPOT (Supporting Methods File S1). This preliminary experiment aimed to select the concentration of TH+SC for use in immunogenicity studies. Nanopatches were coated with ChAd63 (A) or MVA (B) either without TH+SC, or with TH+SC at concentrations of 5% w/v total disaccharide (equating to approximately 0.15M, as per [23]) or 10% w/v total disaccharide (maximum concentration possible without negatively affecting coating morphology). Results indicated that of the two concentrations, optimal ChAd63 immunogenicity was induced with addition of 10% w/v TH+SC, whereas inclusion of TH+SC to MVA vaccines was not a requirement for the induction of a response. Therefore, 10% w/v TH+SC was taken forward as the optimal concentration for ChAd63 stabilisation. (DOCX) [file pone.0067888.s001.docx]
