# Supplementary material for: Dry-Coated Live Viral Vector Vaccines Delivered by Nanopatch Microprojections Retain Long-Term Thermostability and Induce Transgene-Specific T Cell Responses in Mice
Source: PLoS One. 2013 Jul 9;8(7):e67888. doi: 10.1371/journal.pone.0067888 (PMC3706440; doi:10.1371/journal.pone.0067888)
Supplement: Table S1 — Proportions of vaccine-containing coated material delivered to the mouse ear skin upon NP application. NP (n = 5) were coated with formulations containing MVA (2.5×10^7^ PFU or ChAd63 (2.5×10^9^ VP) with or without the addition of 10% w/v TH+SC. 4 nCi of the radio label 14C-Ovalbumin (14C-OVA). Coated NP were delivered to mice and β particle emission detected from excised ears, swabs used to remove material on the ear surface and used NP. Proportions of 14C-OVA detected by scintillation assay present within the excised ear, as a percentage of the total 14C-OVA (ear+swab+NP) are given in the table. The summed counts were similar to elution controls – NP coated and immediately eluted into PBS (not shown). The delivery efficiencies of ChAd63.ME-TRAP and MVA.PbCSP were similar (ChAd63; 16.5% ±2.8, MVA; 14.5% ±4.0).When TH+SC were added into the formulation, the delivery efficiency was significantly reduced (P<0.0001), though scintillation counts were significantly higher than the limit of detection (averaged counts from 10 vials of PBS only, not shown). Delivery efficiencies of vectors with TH+SC addition were 1.3% ±1.1 (ChAd63) and 0.08% ±0.06 (MVA). The addition of TH+SC significantly increased the viscosity of the coating solution (empirical observation). Consequently, we speculate that the low delivery efficiency of virus when formulated with TH+SC is due to imperfect coating morphology mediated by increased viscosity of coating solution. (DOCX) [file pone.0067888.s002.docx]

# Supporting Information Table S1 – Pearson et al

| \| **Formulation (+MC +PS20)** \| **Percentage of coated ^14^C-OVA detected in ear skin (± SD)** \| \| --- \| --- \| \| MVA.PbCSP \| 14.5 ± 4.0*** \| \| MVA.PbCSP + TH+SC \| 0.08 ± 0.06 \| \| ChAd63.ME-TRAP \| 16.5 ± 2.8*** \| \| ChAd63.ME-TRAP + TH+SC \| 1.3 ± 1.1 \| |  |  |  |  |  |
| --- | --- | --- | --- | --- | --- | --- | --- | --- | --- | --- | --- | --- | --- | --- | --- |
|  |  |  |  |  |  |
