# Supplementary material for: Dry-Coated Live Viral Vector Vaccines Delivered by Nanopatch Microprojections Retain Long-Term Thermostability and Induce Transgene-Specific T Cell Responses in Mice
Source: PLoS One. 2013 Jul 9;8(7):e67888. doi: 10.1371/journal.pone.0067888 (PMC3706440; doi:10.1371/journal.pone.0067888)
Supplement: Table S2 — Table listing all patents filed on the Nanopatch technology and details of author involvements. (DOCX) [file pone.0067888.s003.docx]

# Supporting Information Table S2 – Pearson et al

| **Title** | **Patent Application No.** | **Status** | **Inventors/contributors (authors)** |
| --- | --- | --- | --- |
| Delivery Device | US 11/496053 | Granted | MAFK |
| Continuation of US 11/496053 (Device) | US 13/251891 | Filed | MAFK |
| Continuation of US 11/496053 (Method) | US 13/251920 | Filed | MAFK |
| Coating Method 1 (Gas jet) | AU 2008341030 | Filed | MAFK, XC, GF |
|  | EP 08865041.1 | Filed | MAFK, XC, GF |
|  | US 12/810298 | Examination | MAFK, XC, GF |
|  | CA 2745339 | Filed | MAFK, XC, GF |
| Manufacture | AU 2009212106 | Filed | MAFK |
|  | CA 2749397 | Filed | MAFK |
|  | CN 200980104635.3 | Filed | MAFK |
|  | EP 09707729.1 | Filed | MAFK |
|  | IN 5655/DELNP/2010 | Filed | MAFK |
|  | US 12/866717 | Filed | MAFK |
| Coating Method 2 (Dip) | AU 2009304594 | Filed | MAFK, XC, GF |
|  | CN 2008905364 | Filed | MAFK, XC, GF |
|  | EP 09820115.5 | Filed | MAFK, XC, GF |
|  | IN 2927/DELNP/2011 | Filed | MAFK, XC, GF |
|  | US 13/124109 | Filed | MAFK, XC, GF |
|  | CA (tba) | Filed | MAFK, XC, GF |
| Dissolving | AU 2009329806 | Filed | XC, MAFK, GF |
|  | CN (tba) | Filed | XC, MAFK, GF |
|  | EP 09833918.7 | Filed | XC, MAFK, GF |
|  | IN (tba) | Filed | XC, MAFK, GF |
|  | US 13/141667 | Filed | XC, MAFK, GF |
|  | CA (tba) | Filed | XC, MAFK, GF |
| Applicator | PCT/AU2011/000890 | Filed | MC, MAFK |
| Delivery device (2) | AU2011904174 | Filed | MC, MAFK |
