# Supplementary material for: Dry-Coated Live Viral Vector Vaccines Delivered by Nanopatch Microprojections Retain Long-Term Thermostability and Induce Transgene-Specific T Cell Responses in Mice
Source: PLoS One. 2013 Jul 9;8(7):e67888. doi: 10.1371/journal.pone.0067888 (PMC3706440; doi:10.1371/journal.pone.0067888)
Supplement: Methods File S1 — Additional materials and methods used within these studies. (DOCX) [file pone.0067888.s004.docx]

# Supporting Methods File S1 – Pearson et al

## Viral viability assays

Details of assays used to determine viral viability after mixing with coating excipients or after dry-coating are below. For elution: dry-coated Nanopatches were added to 1 mL Dulbecco’s Modified Essential Medium + 10% ^v^/_v_ Foetal Bovine Serum (FBS) + 100 U Penicillin, 0.1 mg/mL Streptomycin + 0.1% ^v^/_v_ L-Glutamine, ‘D-MEM’ (all Sigma-Aldrich).

## ChAd63 hexon immunostaining

Human Embryonic Kidney 293A cells (Invitrogen) were seeded in 24-well plates and allowed to reach confluency. Serial dilutions of ChAd63-containing coating formulations or eluates were made in D-MEM and incubated with cell monolayers for 48 hours (37 °C + 5% CO_2_). Negative control wells contained cells + D-MEM only. Following incubation, D-MEM was replaced with 500 µL cold methanol. After 24 hours at -20 °C, immunostaining of the Ad hexon protein was carried out using a QuickTiter^TM^ Adenovirus Immunoassay kit (Cell Biolabs Inc, CA, USA) according to instructions. Cells infected with virus were visualised under light microscope and counted in 5 fields of view per well. Recovered viral titre was calculated as infectious units (IFU) per mL.

## MVA plaque assay

DF-1 cells (ATCC ref. CRL-12203) were seeded in 6 well plates and were allowed to reach confluency. MVA.GFP formulations and eluates were serially diluted and incubated with cell monolayers. After 1 hour, 1:1 carboxymethyl cellulose + D-MEM with 2% FBS was overlaid. After incubation for 3 days at 37 °C, MVA plaques were counted under fluorescent microscope (Leica, Germany). Viability was calculated as Plaque Forming Units (PFU)/mL.

## Assessment of Immunogenicity by IFN-γ ELISPOT

ACK lysis buffer-treated PBMC or splenocytes were re-suspended in Minimal Essential Medium (α-modification, Sigma-Aldrich) + 10% ^v^/_v_ FBS, 100 U Penicillin + 0.1 mg/mL Streptomycin, 4 mM L-Glutamine (all Sigma-Aldrich). Cells were incubated for 18 hours at 37 °C with peptide Pb9 (SYIPSAEKI) at a final concentration of 1µg/mL on anti-IFN-ɣ (AN18) pre-coated nitrocellulose membrane plates (Millipore, UK). Pb9 is an MHC class I (H-2K^d^) epitope is common to both PbCSP and ME-TRAP transgenes. Naive splenocytes (5x10^6^ per mL) were added to wells containing PBMC to enhance antigen presentation. Following incubation, plates were developed by 1 hour incubations with anti-IFN-γ biotin conjugated antibody and Streptavidin-Alkaline Phosphatase (Mabtech, Sweden). Alkaline Phosphatase development buffer was added according to kit instructions (Biorad, UK). Spots were counted using an AID plate reader and software. Spot Forming Cells (SFC) per million cells was calculated for each sample using cell count taken before plating.

## Estimation of delivery efficiency by radioassay

Ovalbumin labelled with radioactive ^14^carbon (^14^C-OVA) was mixed with formulations containing vaccine. Each coated NP contained 4 nCi ^14^C-OVA. After NP application to mouse ear skin, ear surfaces were swabbed 3 times with a PBS-wetted cotton bud. Cotton buds, used NP and ears excised from euthanised mice were collected into scintillation vials (Thermoscientific, USA) and 1 mL PBS (swabs, patches) or 1 mL tissue solubiliser solution (Solvable®, Perkin Elmer) added. Ear samples were incubated at 60 °C overnight. After vortexing, 10 mL of scintillation fluid (Ultima Gold^TM^, Perkin Elmer) was added to each vial before β-particle emission was detected using a PerkinElmer TriCarb 2810 TR liquid scintillation analyser.
